# Flexible Delivery Patch Systems based on Thermoresponsive Hydrogels and Submicronic Fiber Heaters

**DOI:** 10.1038/s41598-018-35914-2

**Published:** 2018-12-03

**Authors:** Alexandru Evanghelidis, Mihaela Beregoi, Victor C. Diculescu, Andrei Galatanu, Paul Ganea, Ionut Enculescu

**Affiliations:** Multifunctional Materials and Structures Laboratory, National Institute of Materials Physics, Atomistilor Street, Magurele, Bucharest 077125 Romania

## Abstract

This paper proposes a novel, flexible, low cost administration patch which could be used as a non-invasive, controlled transdermal drug delivery system. The fabricated device consists in a flexible microfiber architecture heater covered with a thermoresponsive hydrogel, namely poly(N-isopropylacrylamide), as a matrix for the incorporation of active molecules. The manufacturing process consists of two main steps. First, the electrospun poly(methyl methacrylate) fiber networks are sputter coated with a thin gold layer and attached to flexible poly(ethylene terephthalate) substrates to obtain the heating platforms. Second, the heaters are encapsulated in poly(ethylene terephthalate) foils and covered with poly(N-isopropylacrylamide) hydrogel sheets. In order to illustrate the functionality of the fabricated patch, the hydrogel layer is loaded with methylene blue aqueous solution and is afterwards heated via Joule effect, by applying a voltage on the metalized fibers. The methylene blue releasing profiles of the heated patch are compared with those of the unheated one and the influence of parameters such as hydrogel composition and morphology, as well as the applied voltage values for microheating are investigated. The results indicate that the fabricated patch can be used as a drug administration instrument, while its performance can be tuned depending on the targeted application.

## Introduction

Wearable electronics, in particular transdermal drug delivery devices represent an important pylon in non-invasive medical care^1^. Even if transdermal administration presents many advantages avoiding gastric and hepatic injuries, only few patches are commercially available^2^. A proper drug delivery patch should be flexible, soft, easy to handle, painless and it should release the drug in a controlled manner. Over the past years, multiple external stimuli have been used for activating the release of a therapeutic agent from their carrier, namely mechanical stress, light, pH, heat, etc.^3–10^. However, the most attractive method for releasing active molecules is heating a thermoresponsive structure, since the heat will not only promote the release of drugs, but it could also enhance skin penetration by causing the pore dilation^11,12^.

A thermoresponsive material frequently used in drug delivery applications is poly(N-isopropylacrilamide) based hydrogel (PNIPAM). It can be synthesized using uncomplicated preparation methods, has great availability of starting materials and it offers the possibility to retain a drug solution at low temperature and to release it by heating up at the body temperature^13^. This behavior is driven by the swelling/shrinking processes which occur in the hydrogel when the temperature is changed, which makes it suitable for use in drug delivery systems. An important hydrogel feature for drug delivery systems is its morphology, as a great porosity will facilitate the drug solution’s absorption/release^14^. In the case of PNIPAM this parameter can be easily controlled by choosing suitable synthesis conditions^15–18^.

In the context of wearable electronics, electrospinning technique can play a key role by allowing the fabrication of fibers with submicron diameters^19^. Metallization of these fibers allow their utilization as microheaters via Joule effect, while their specific microscopic structure increases the heating efficiency^20–22^. Although their conductive properties have been studied before, the spatial distribution of power dissipation through nonwoven fiber networks created through electrospinning has not been investigated so far. There is only one study which deals with the sheet resistance of nonwoven metallic fabrics, but it does not discuss spatial distribution, this parameter being notably important for controlled heating applications^23^.

This work presents the fabrication of a fully functional wearable delivery patch which could be employed as a controllable transdermal drug administration tool. The patch’s manufacturing process involves two main steps. First, a poly(methyl methacrylate) (PMMA) solution was electrospun to obtain solid fibers with submicron sizes. The fiber networks were covered with a thin gold layer in order to obtain a conductive path, which under applied voltage generates heat. To improve their mechanical properties while maintaining flexibility, the metalized meshes were thermally attached to poly(ethylene thereftalate) (PET) substrates. In the second step, the heating platform encapsulated in PET foils was covered with a PNIPAM layer. The functionality of the proposed patch was demonstrated through the controlled release of methylene blue (MB) as an electrochemically active compound^24^. The MB releasing profiles were registered when the hydrogel coated patch was heated under applied voltage. A theoretical model for simulating the temperature distribution through fibers was proposed and confirmed by thermographic images captured under applied voltage, and the hydrogel morphology and composition, as well as the kinetic of MB release, were investigated. The obtained results indicate that the fabricated patch can be used as a drug administration instrument for applications in which the release process should be fast and precise.

## Results and Discussion

### Structural characterization of PNIPAM

**Figure 1:**
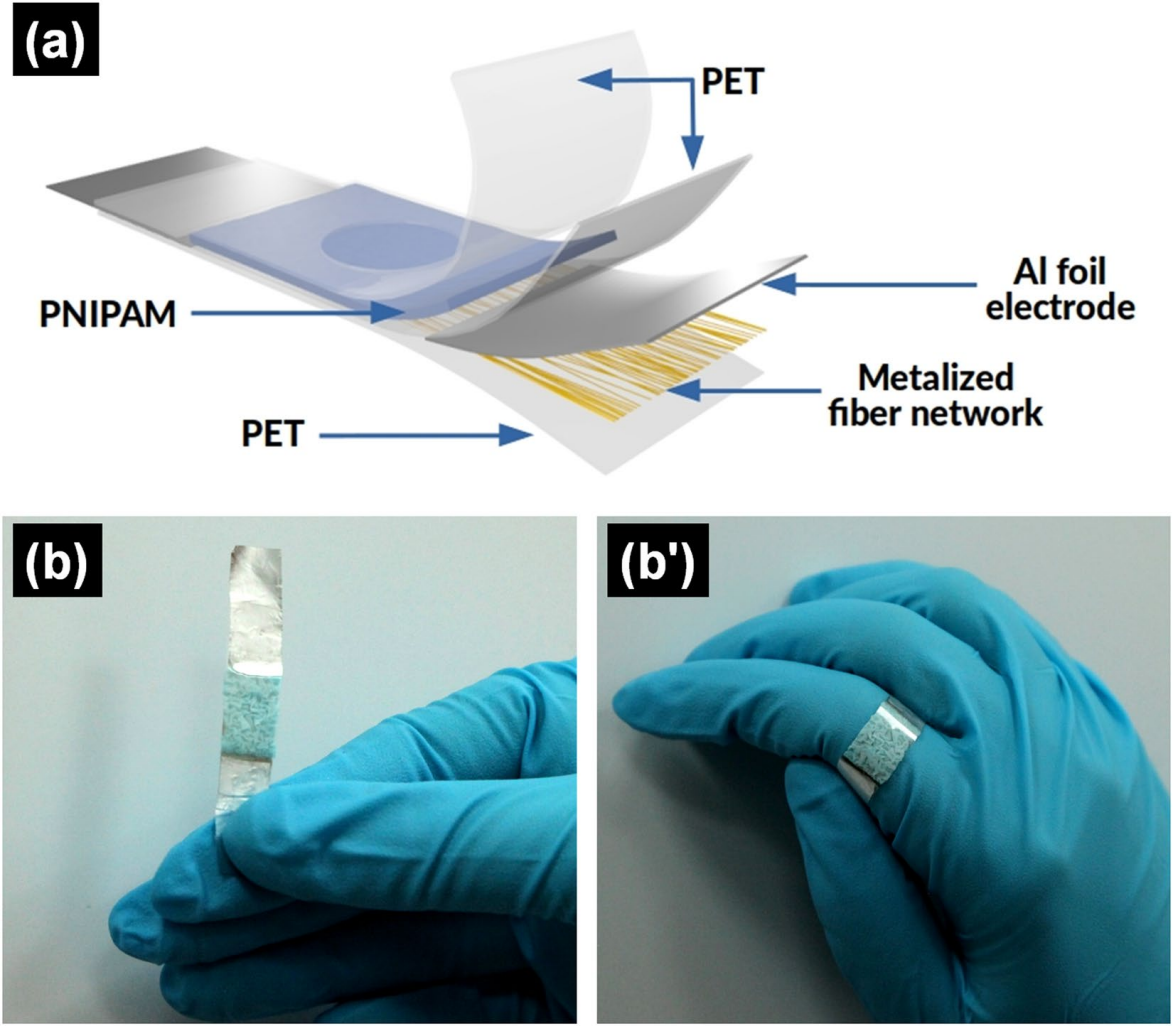
(**a**) Schematic representation and (**b**,b’) digital photographs of the microheated patch.

A schematic representation of the components of the flexible patch is presented in Fig. 1a and digital photographs of the manufactured patch covered with the thermoresponsive hydrogel is shown in Fig. 1(b,b’).

**Figure 2:**
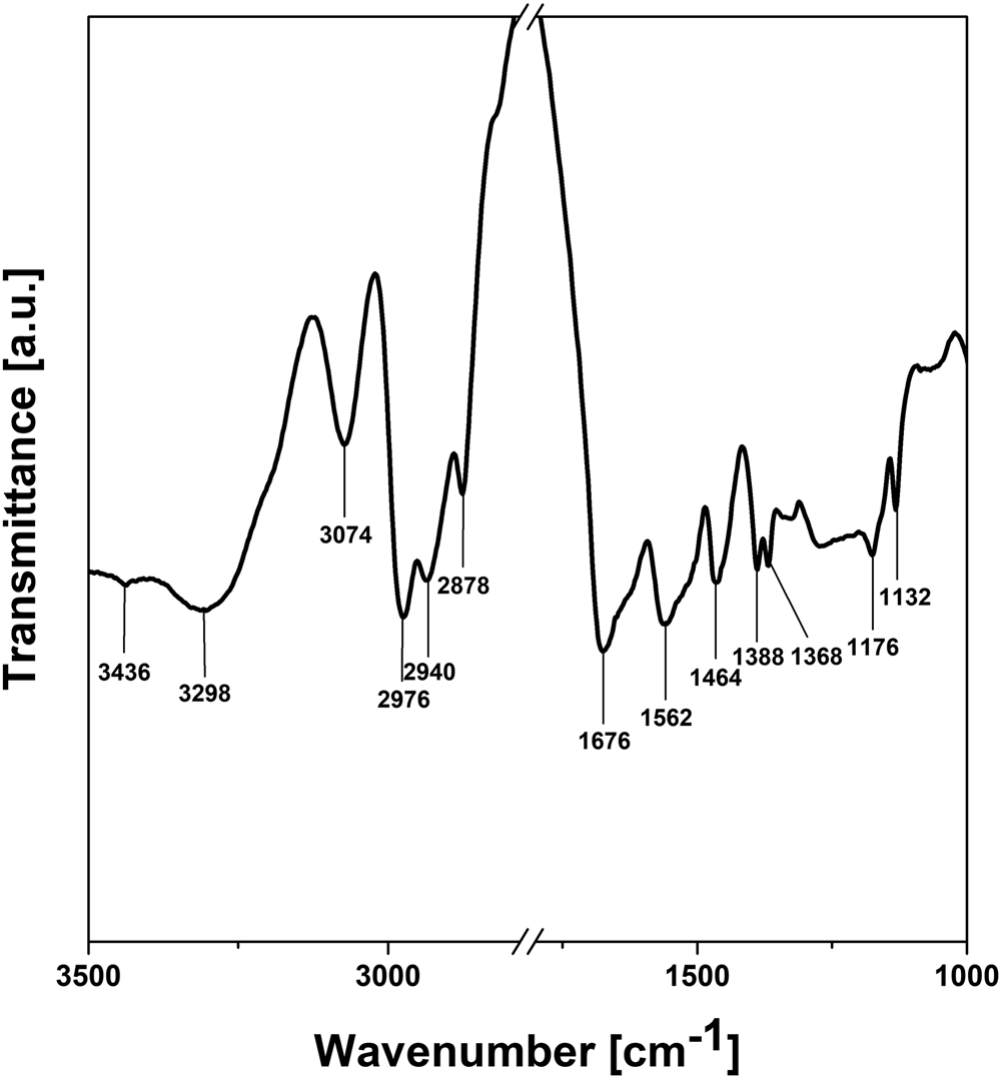
FTIR spectrum of PNIPAM hydrogel crosslinked with MBA.

The FTIR spectrum of PNIPAM crosslinked with MBA is depicted in Fig. 2. It can be noticed the characteristic absorption bands of this material: the absorption peaks at 3436, 3298 and 3074 cm^−1^ are related to the N-H stretching in the secondary amides, 2976, 2940 and 2878 cm^−1^ are assigned to the asymmetric and symmetric stretching vibration of C-H bonds, 1676 cm^−1^ is attributed to C=O stretching mode of amide I, 1562 cm^−1^ to N-H stretching vibration of amide II and 1464 cm^−1^ to CH_3_ asymmetric deformation. As well, the absorption bands at 1388 and 1368 cm^−1^ are characteristic to the symmetric deformation of –C(CH_3_)_2_ group, 1176 and 1132 cm^−1^ being assigned to the CH_3_ vibration mode^25,26^.

### Morphological analysis of PNIPAM

**Figure 3:**
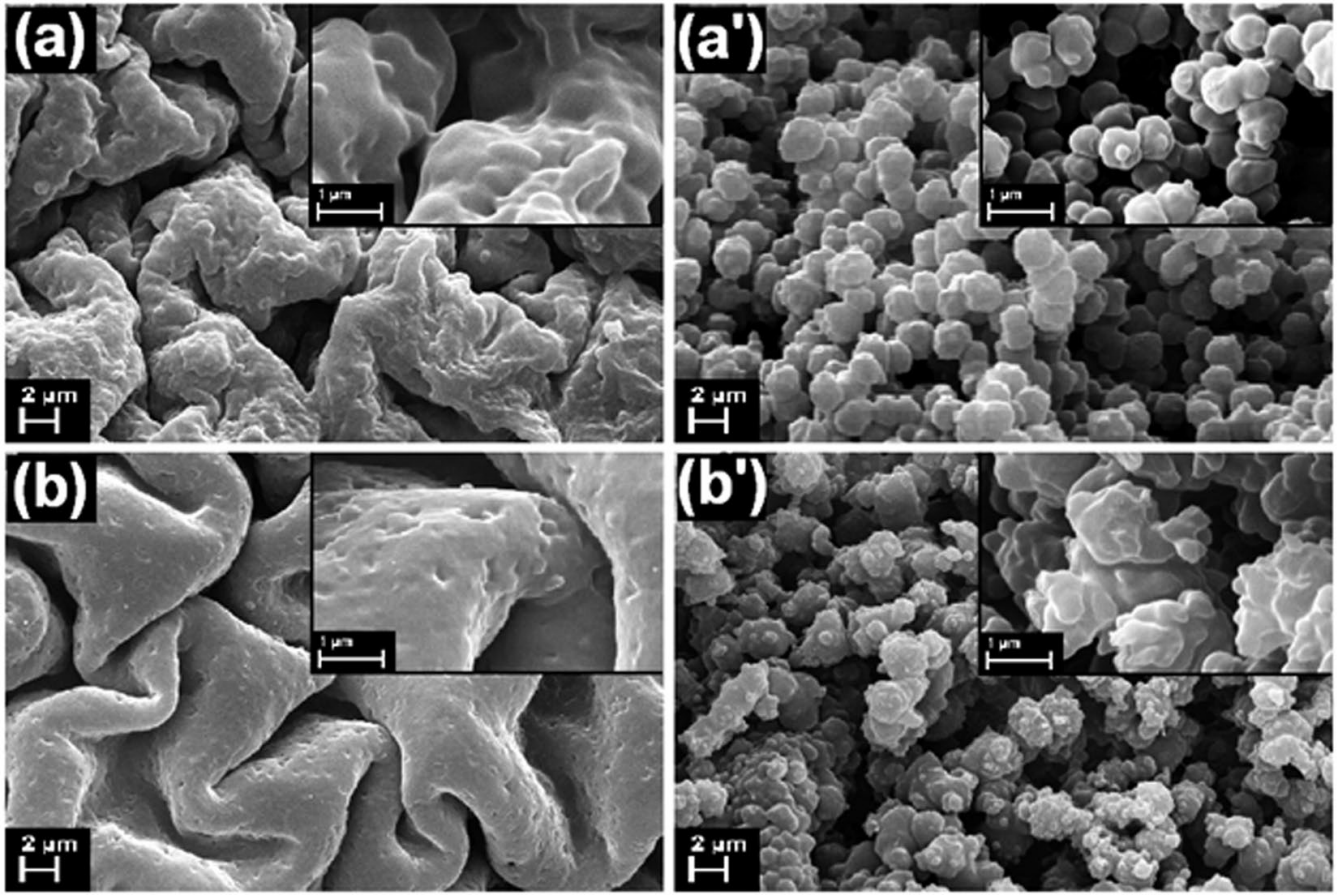
SEM images at high (inset) and low magnification of (**a**,a’) P_1_ and (**b**,b’) P_2_, on (**a**,**b**) top and (a’,b’) in cross section of the hydrogels, respectively.

It is well known that the crosslinking degree and the concentration of the monomer, hence the morphology, influences the swelling profile of the prepared hydrogel. For this reason, two hydrogel compositions (P_1_ and P_2_) were prepared and the materials were morphologically characterized by SEM after dehydration to dryness. Figure 3 shows the SEM images of samples P_1_ and P_2_ taken on top (Fig. 3(a,b)) and in cross section (Fig. 3(a’,b’)). The surface of the hydrogels presents a brain-like structure, the porosity and the size of the convolutions raising when the NIPAM concentration is increased. The cross section analysis reveals that the P_1_ hydrogel has a granular morphology with rare, spherically-shaped micrograins with a uniform distribution of sizes. Contrary, the P_2_ hydrogel presents grains with various sizes, being more dense and interpenetrated, diminishing in this way the porosity of the material. As it was expected, the hydrogel composition is well correlated with the material morphology, which further influences the swelling/releasing profile, thus the insertion and the removal of MB solution in/out of P_2_ will be more difficult to achieve.

### Heating performances of metalized fiber networks

Aside from power consumption, an important aspect regarding the performance of a material in heating applications is how uniform it distributes the energy over an area. Thus, it was investigated whether the discrete and disordered nature of metalized fiber webs would have a significant influence on the spatial distribution of power dissipation by simulating an idealized fiber network as an electronic circuit.

As disordered microstructured materials are often exploited both for their transparency, not just flexibility, optical transmission is a useful measure for comparison between samples. In plain fiber webs, transmittance is a function of web density, with denser webs being more opaque. This can be explained as an increase in diffuse scattering due to larger numbers of otherwise quasi-transparent polymer fibers which intersect and overlap randomly. Adding a metallic layer increases opacity by effectively blocking any light passing through the fibers and adding a reflection factor. In this case, a simplified geometrical shadow loss model for transmittance can be used, where the entire web is treated as a planar aperture with a certain fraction of its total area covered by completely opaque subdomains. Transmittance, then, is merely the percentage of the total area of the sample which is not covered by fibers.

**Figure 4:**
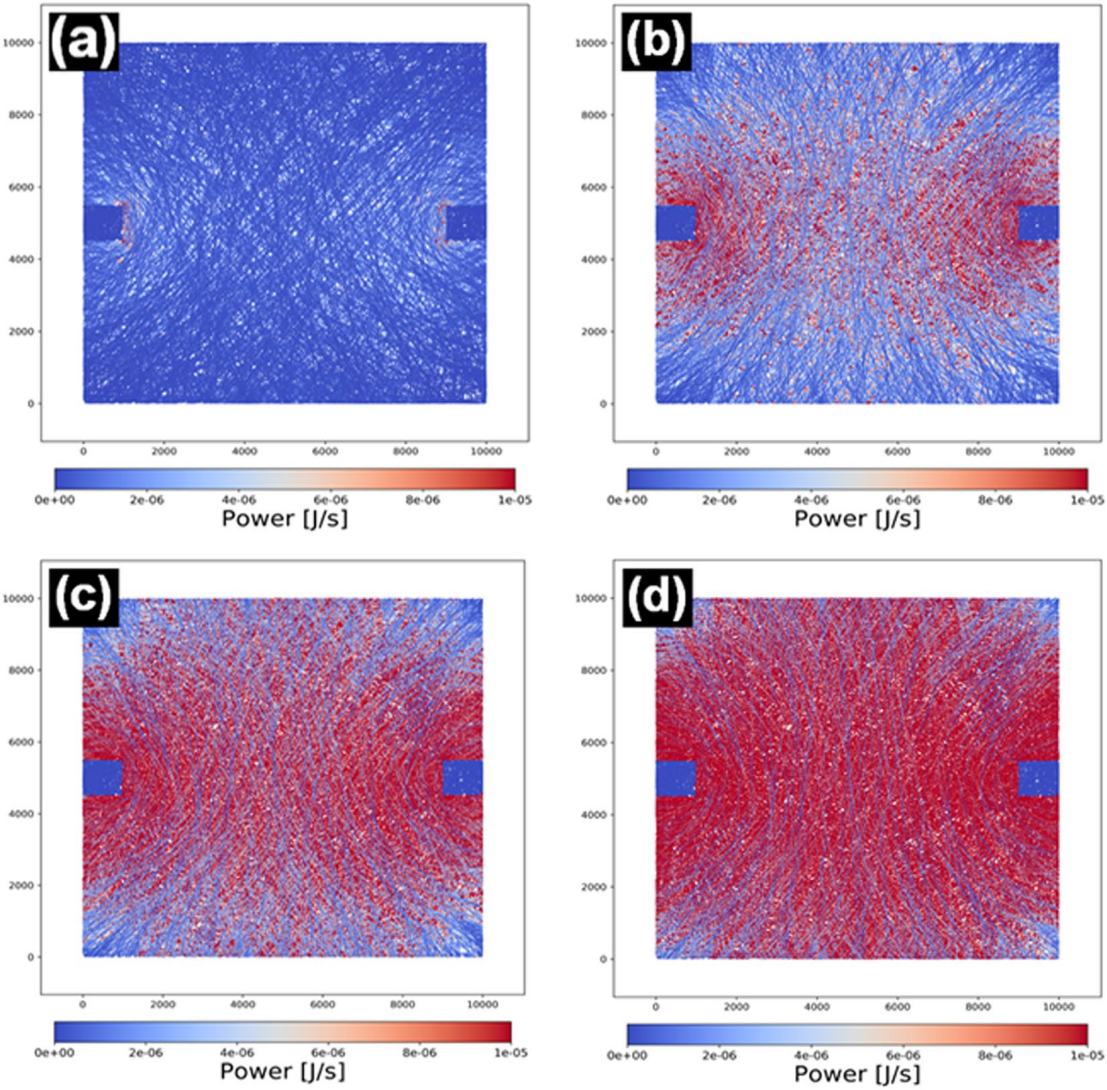
Heating maps of metalized fiber network electrode resulted from the theoretical simulation process at (**a**) 1 V (**b**) 3 V (**c**) 5 V and (**d**) 8 V applied voltage.

The evolution of the power distribution in a simulated metalized fiber network as voltage is increased is presented in Fig. 4. It can be noticed that at a macroscopic scale, the material behaves as if continuous, showing a typical spatial distribution of power and, implicitly, electric field. An interesting feature that can be observed at smaller voltages (1 and 3 V, Fig. 4a,b) is that the current flows mostly through a relatively small number of fibers which directly connect the electrodes. Even at higher voltages (5 and 8 V, Fig. 4c,d, respectively), it can be seen how some of the fibers, more or less perpendicular to the imaginary line drawn by the electrode placement, don’t contribute at all.

**Figure 5:**
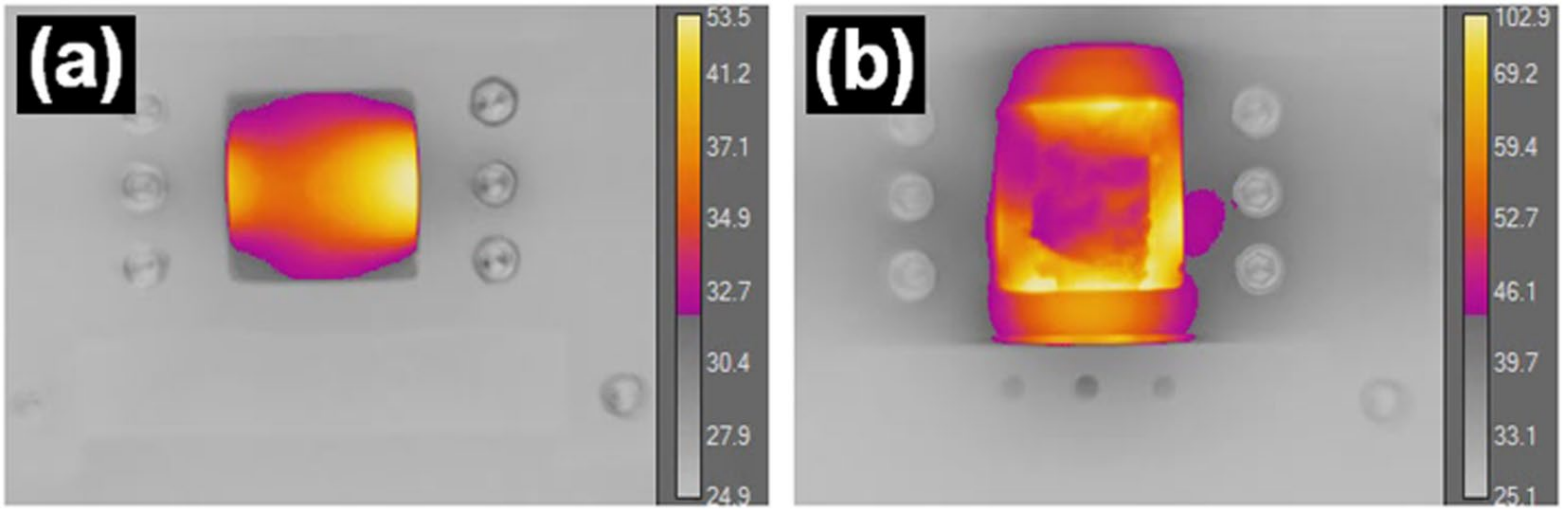
Thermographic images of (**a**) uncovered and (**b**) PNIPAM hydrogel covered metalized fiber network electrode at 8 V applied voltage.

These results are corroborated with thermographic images of an actual physical sample in a similar electrode configuration (Fig. 5a), which show how the heat distribution in the metalized fiber network has the same overall shape as the power dissipation map. A thermographic image of a sample covered with PNIPAM hydrogel (Fig. 5b) during heating was also taken.

The numerical model is calculated in a static regime, i.e. it considers only the instantaneous values for voltage and current and does not take into account the effects of heating on resistance. A reasonable concern would be that, as an individual fiber segment carries current and heats up, the resulting increased resistance would cause less current to flow through it, through the nearby paths of lesser resistance. Since the large surface-to-volume ratio of the metalized fibers likely leads to rapid cooling, microscale oscillations are likely to occur. Given this, the fact that the resulting power dissipation maps closely resemble the physical heat maps suggests that any oscillations which may take place due to the microstructure of the material do not have a considerable influence on macroscopic heating performance, and/or that a steady state is attained very quickly.

These results are useful for designing heaters based on metalized micro/nanofiber networks, specifically when temperature uniformity across a given surface is required.

### Methylene blue releasing profile

**Figure 6:**
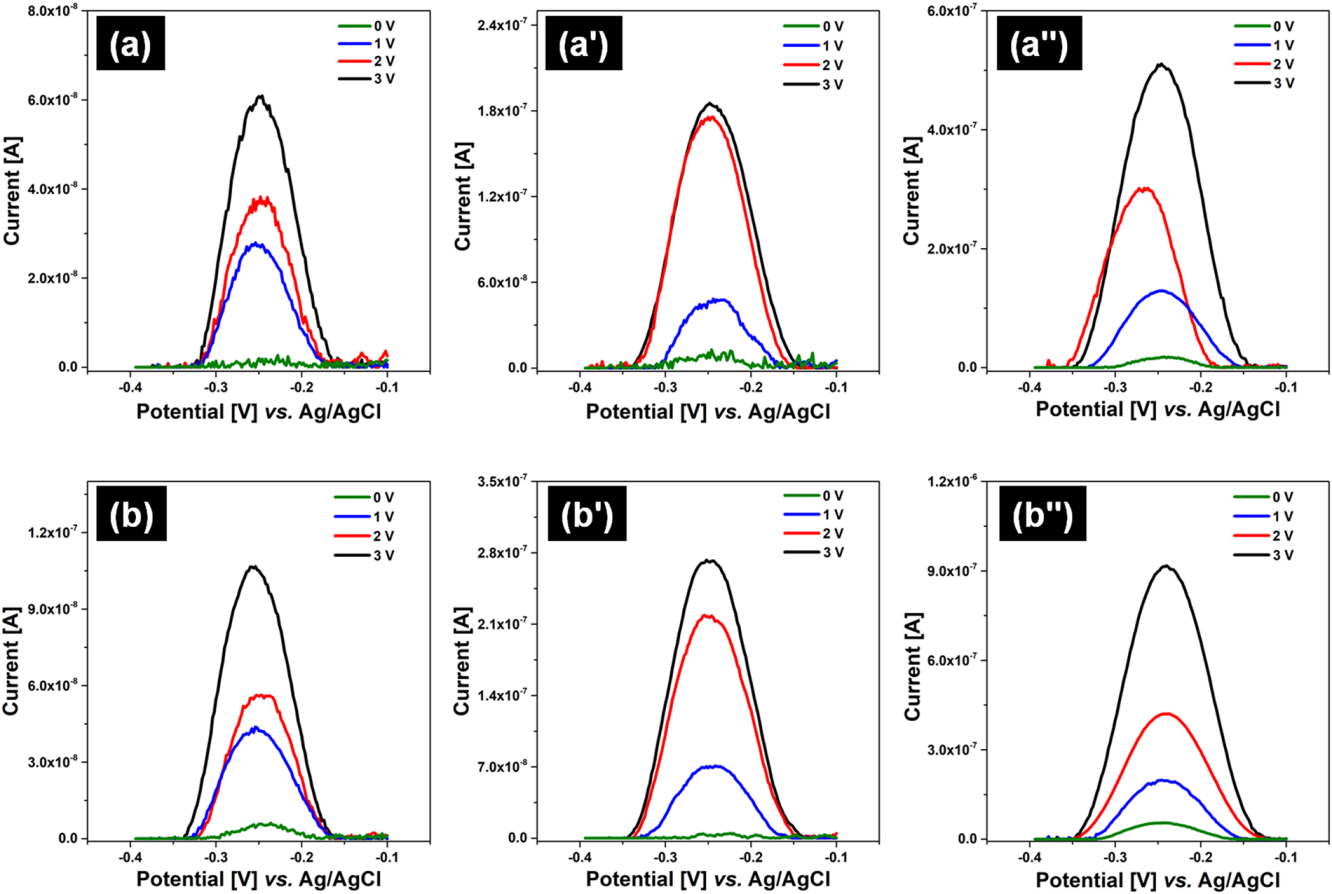
SWVs registered for samples (**a**,a’,a”) P_1_ and (**b**,b’,b”) P_2_, when the hydrogel sheets were swollen in MB loading solutions of (**a**,**b**) 0.25 mM, (a’,b’) 0.50 mM and (a”,b”) 1.00 mM with and without increasing the patch temperature at different heating voltage values.

The functionality of the fabricated patch was highlighted by evaluating the capacity of the device to controlled release an electrochemical active compound on-demand. With this purpose, the microstructured heated patches were covered with the thermoresponsive hydrogel P_1_ and P_2_ sheets which were then swollen in MB loading solutions of various concentrations (0.25, 0.50 and 1.00 mM). The MB releasing profile from P_1_ and P_2_ was evaluated by applying heating voltages of 1, 2 and 3 V for 5 min in order to increase PNIPAM temperature. The hydrogel released MB in a fixed volume of 1 mL PBS solution through the cell aperture, which provides the physical contact between the electrolyte and the hydrogel. Figure 6 shows the SWVs registered using samples P_1_ (Fig. 6(a,a’,a”)) and P_2_ (Fig. 6(b,b’,b”)) for heated and unheated patches. In all SWVs, the peak characteristic to MB was identified at −0.25 V^27^.

In both cases, the peak increases by raising heating voltage values and MB loading solution concentrations. On the other hand, lower currents were observed for P_2_ sample showing that the releasing profile depends on the hydrogel composition.

The experiment described above showed on one hand that increasing the NIPAM concentration, the delivered MB amount decreased, as a consequence of hydrogel morphology. In fact, SEM images proved P_2_ as a lower porosity material, which influences the swelling/releasing profile, thus the penetration and the removal of MB solution in/out of P_2_ is more difficult to take place. Likewise, the increase of the MB loading solution concentration provides a higher MB released quantity. It is also obvious that the heating process improves the MB delivery: without heating, only a small MB concentration was released while application of different heating voltages increases the released MB mass. These results are a consequence of heating the thermoresponsive hydrogel, the temperature increase forcing it to contract, hence expelling incorporated MB molecules from its structure.

**Figure 7:**
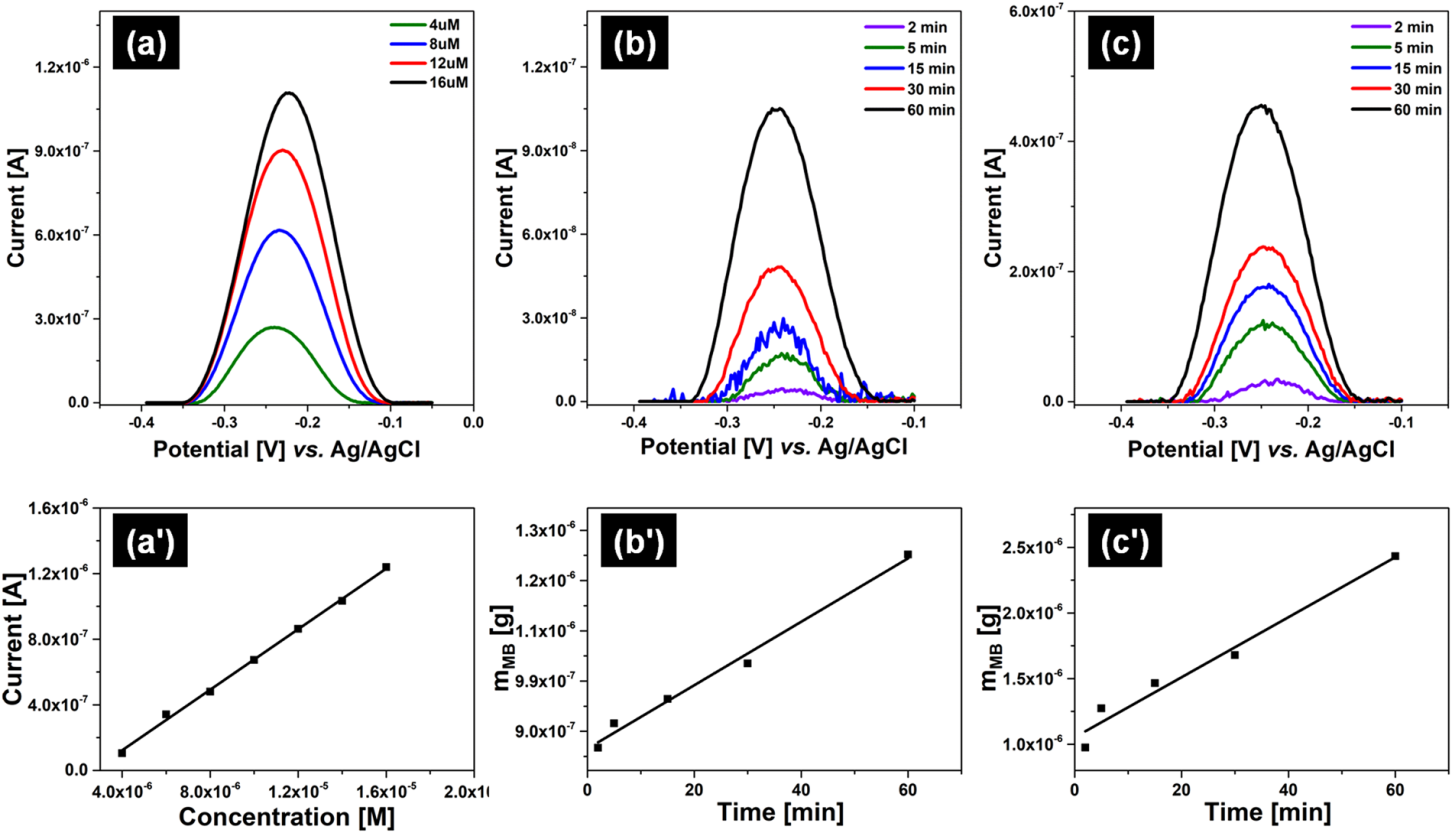
SWVs registered for samples (**a**,a’,a”) P_1_ and (**b**,b’,b”) P_2_, when the hydrogel sheets were swollen in MB loading solutions of (**a**,**b**) 0.25 mM, (a’,b’) 0.50 mM and (a”,b”) 1.00 mM with and without increasing the patch temperature at different heating voltage values.

The kinetic of MB liberation from patches covered with hydrogel sheets was also investigated. With this intent, a MB calibration curve that correlates MB concentration in PBS with the oxidation current was recorded (Fig. 7a). SWVs were registered in solutions of MB with concentration up to 16 µM. Between the experiments, the electrode surface was always cleaned in order to avoid any adsorption interference. Linearity was observed on the whole concentration range following equation 0.0923 ± 0.00182.

The MB releasing profiles from patches covered with P_2_ hydrogel sheets when the patch was unheated (Fig. 7b) and heated (Fig. 7c) was also investigated.

The hydrogels were swollen with 0.50 mM MB loading solution for 30 min, and SWVs were recorded always with a clean GCE surface after different time intervals. Between the voltammetric measurements, the patch was unheated or heated at about 3 V. Irrespective of the applied heating voltage, the peak increased linearly with time in agreement with the MB release from the hydrogel. Nevertheless, higher currents were registered for the heated sample. By using the calibration curve (Fig. 7a’), the recorded currents were transformed into concentration/weight values and the releasing rate was calculated. It was found that by heating the patch, the MB is released four times faster than without heating, thus under applied heating voltage the rate of release being 2.29·10^−8^ g/min MB (Fig. 7b’), compared with 5.68·10^−9^ g/min (Fig. 7c’).

**Figure 8:**
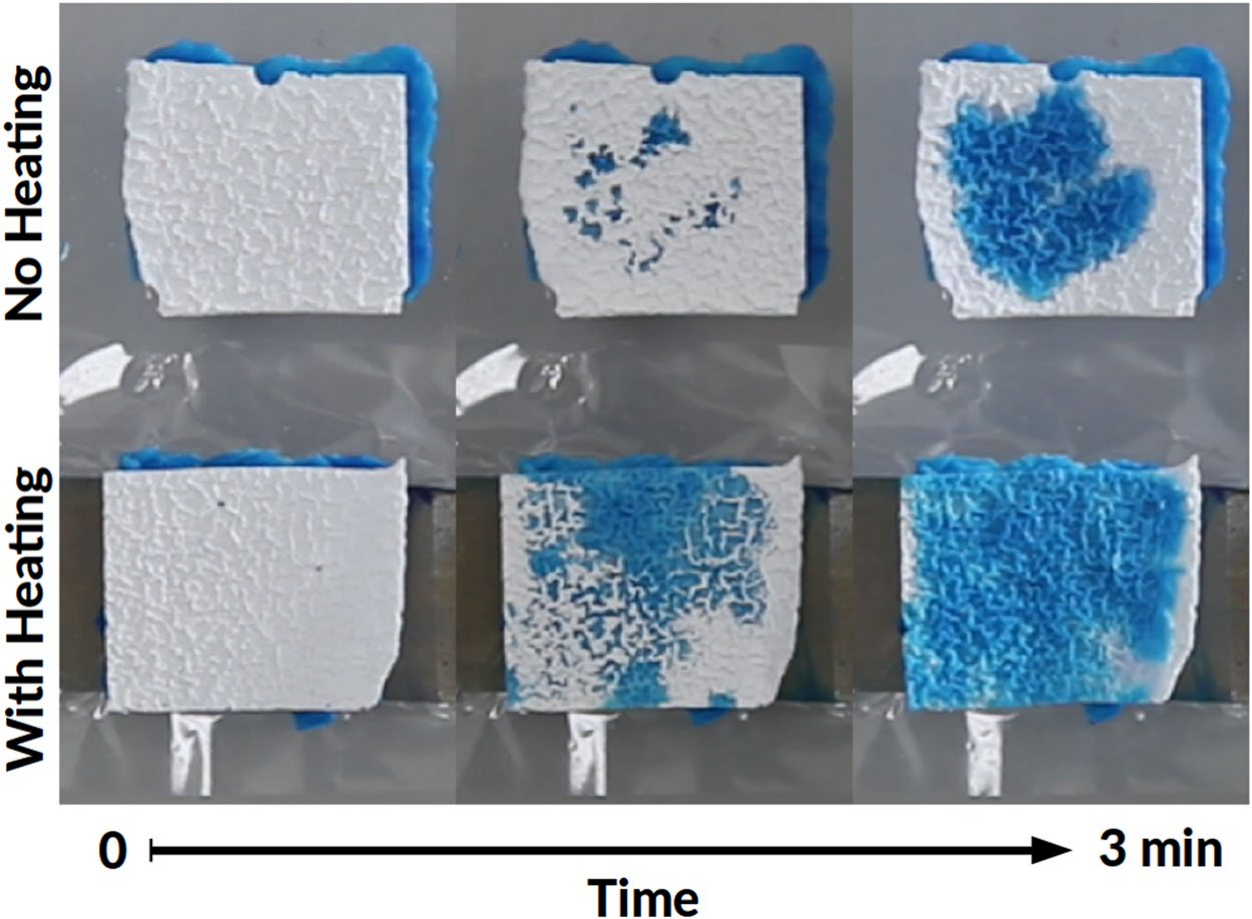
Visual demonstration of the delivery action for a hydrogel patch: unheated (top) and heated (bottom).

To obtain a clear visual demonstration of delivery action, the patch and a separate, similar piece of loaded hydrogel were both covered with thin (~0.34 mm) pieces of unloaded, dry PNIPAM, to act as absorbent and indicator. The patch was turned “on” at a 300 mA level for 2 minutes, until the indicator gel became fully colored and no further evolution was observed. Figure 8 shows the strong influence of heating over the diffusion of the methylene blue solution.

Due to the highly absorbent nature of dry PNIPAM hydrogel, the coloring occurs also in the unheated case as well, but at a much slower pace.

## Conclusion

A novel flexible patch configuration which could be used as a non-invasive, controlled transdermal drug delivery system was developed. Electrospun polymer fiber networks covered with a thin gold layer and attached to flexible PET substrates were employed as microstructured heaters. The power distribution through such conductive fiber networks was simulated with a numerical model and it was found that the simulation results are in good agreement with thermographic images captured during the heating process. A thermoresponsive hydrogel that encapsulates and releases the active compound was employed. The structural and morphological characteristics of the prepared hydrogels were investigated, proving the formation of poly(N-isopropylacrilamide) and identifying its microgranular structure.

The performances of the fabricated patch were tested using a methylene blue solution with different concentrations and by applying up to 3 V to heat the patch. As well, two hydrogel compositions were tested in order to find the best releasing profile. The MB delivery is strongly influenced by the hydrogel composition, which correlates with the morphology, and by the concentration of the MB loading solution, release time and applied voltage. Thus, it was found that the heated patch releases MB four times faster than unheated one due to the shrinking of the PNIPAM hydrogels. Therefore, the obtained results indicate that the manufactured patch is reliable for drug delivery applications and its releasing properties can be adjusted by changing the fabrication parameters.

## Methods

### Materials

Poly(methylmethacrylate) (M_w_ = 350.000 g/mol, Aldrich), N,N-dimethylformamide (≥99.8%, Honeywell), N-isopropylacrylamide (97%, Aldrich), N,N′-methylenebisacrylamide (powder, ≥99.5%, Sigma), 2-hydroxy-4′-(2-hydroxyethoxy)-2-methylpropiophenone (98%, Adrich), methylene blue hydrate (≥97.0%, Sigma) were used as received. Phosphate buffered saline (PBS, bioreagent for molecular biology, pH 7.4 at 25 °C, Sigma) was prepared by dissolving the content of one PBS packet (NaCl 0.138 M and KCl 0.0027 M) in one liter of water. All solutions were prepared using analytical grade Millipore deionized water.

Poly(ethylene terephthalate) foils having a thickness of 0.038 and 0.006 mm were purchased from Good Fellow. Commercial aluminum foil was used for the electrical contacts.

### PNIPAM hydrogels synthesis

PNIPAM hydrogels were chemically synthesized by copolymerizating of N-isopropylacrylamide (NIPAM) with N,N′-methylenebisacrylamide (MBA), the polymerization being initiated by a UV initiator, 2-hydroxy-4′-(2-hydroxyethoxy)-2-methylpropiophenone (Irgacure 2959). For comparison purpose, two hydrogel compositions were prepared by changing the monomer concentration and keeping constant the rest of the experimental parameters: for sample P_1_, the NIPAM concentration being 20% (w/v), while for P_2_, being 30% (w/v). In both cases, the molar ratio between NIPAM: MBA was 50: 1 and the concentration of the initiator was 0.8% (w/v). The monomer, crosslinker and initiator were dissolved in deionized water and the prepared solutions were photoiradiated using a UV lamp (254 nm) in order to obtain solid hydrogel sheets. Further, the samples were rinsed with deionized water to remove any unreacted compounds and dried over night in ambient conditions.

### Fabrication of PNIPAM hydrogel coated microheated patch

The microheated patch coated with PNIPAM hydrogel was fabricated considering the following steps. First, microheaters in a metalized electrospun fiber network configuration were prepared via electrospinning. A PMMA in N,N-dimethylformamide (DMF) solution with a concentration of 10% (w/v) was electrospun in ambient conditions. The PMMA fibers were collected on copper frames placed at a distance of 20 cm from the spinneret. The electrospinning parameters were chosen so that the fibers were uniformly distributed on the frames, with an optimal density. The applied voltage on the spinneret was 20 kV, the solution flow rate being 0.2 mL/h and the collection time 2 minutes. In order to obtain conductive microfibers, the electrospun meshes attached to copper frames were sputter covered with a thin gold layer (200 nm). To improve the mechanical properties, the metalized PMMA fibers were thermally attached to flexible PET substrates by heating the ensemble at 150 °C.

Further, flexible heaters with preset sizes were covered with a thinner PET foil to protect the electronic platform from humidity, avoiding in this way unwanted reactions which could take place during the electrochemical measurements. The extremities of the heater were covered with aluminum foils to achieve a reproducible electrical contact. The prepared PNIPAM hydrogel sheets were immersed for 30 min in MB aqueous solution with different concentrations (0.25, 0.50 and 1.00 mM) and were then placed on the encapsulated heaters. Another PET foil with an aperture that provides the contact with the outside environment (e.g. electrolyte, skin, etc.) was placed on the fabricated device.

### Characterization

Fourier Infrared Spectroscopy (FITR) spectrum of the dried hydrogel was registered using a Perkin Elmer Spotlight Spectrum 100 Spectrometer. Morphology of the prepared hydrogels and metalized fiber networks was analyzed using a Zeiss Evo 50 XVP Scanning Electron Microscope (SEM). The thermographic images were recorded using a “FLIR A305sc” with an IR resolution of 320 × 240 pixels and “ResearchIR” software for control and analysis camera while up to 8 V were applied (in order to generate the heat) on metalized fiber networks attached to glass substrates covered or not with hydrogel sheets. The digital photographs were taken using a Canon DS126201 professional camera.

The electrochemical investigations employed for determining the release parameters were performed using an Ivium Potentiostat/Galvanostat with a homemade electrochemical cell that consists of a conically-shaped upper part and a bottom part. The conical-shaped upper part of the cell presents an aperture (3 mm diameter), which allows a controlled contact between the hydrogel surface and the electrolyte enabling therefore the transfer of MB. The releasing profile of MB was examined considering that the hydrogel was in contact with the electrolyte only in the aperture area. The bottom part of the electrochemical cell perfectly seals the entire assemble and its surface contains the hydrogel covered patch in such way that the aluminum contacts of the ensemble were left outside in order to avoid secondary reactions, which could appear under applied voltage. The used electrodes were: a glassy carbon electrode (GCE) with an active area of about 0.026 cm^2^ as working electrode, a Pt/Ti wire as counter electrode and commercial Ag/AgCl as reference. The electrolyte was 1 mL PBS with pH 7.4. Square wave voltammetry (SWV) was registered by scanning the potential between −0.40 and +0.10 V at 25 Hz frequency and 2 mV step potential for *v* = 50 mV s^−1^, and a pulse amplitude of 50 mV.

Different applied voltages (up to 3 V) to heat the thermoresponsive hydrogels were also considered. It has to be mentioned that the applied voltages were chosen in such a way that the recorded current was fixed at 100, 200 and 300 mA, further increasing the applied voltage causing the fibers to break down. In all the cases, the voltage needed to heat the hydrogel was applied using a SPD3303S Siglent DC power supply. The calibration curve was estimated registering the SWVs for MB aqueous solutions with default concentrations, using the same cell configuration as for MB releasing tests, being presented as a statistic for five measurements (Standard Deviation: 1.92596·10^−8^).

### Theoretical simulation details

The work flow was adapted from a similar approach applied to nanowire networks, and consisted of three main steps: generating a random web, creating a graph with fiber intersections as nodes connected by edges weighted according to distances and using that graph as basis for an electronic circuit simulation^28^. The algorithm used to generate the web is fairly straightforward. At every iteration, two points are chosen at random on opposing sides of a square and their coordinates are stored in an indexed list of lines. The distance between them is multiplied with a set width, thus obtaining the area of the fiber, which is added to a total fiber area. An intersection check is made for each of the previous lines and, if positive, the coordinates of the point of intersection and the indexes of both lines are stored in a list of junctions. The overlap area of each intersection is also calculated and subtracted from the total fiber area. The program iterates until the total area of the fiber reaches a set value, given as a percentage of the total area of the square, i.e. until the desired transmission is achieved.

**Figure 9:**
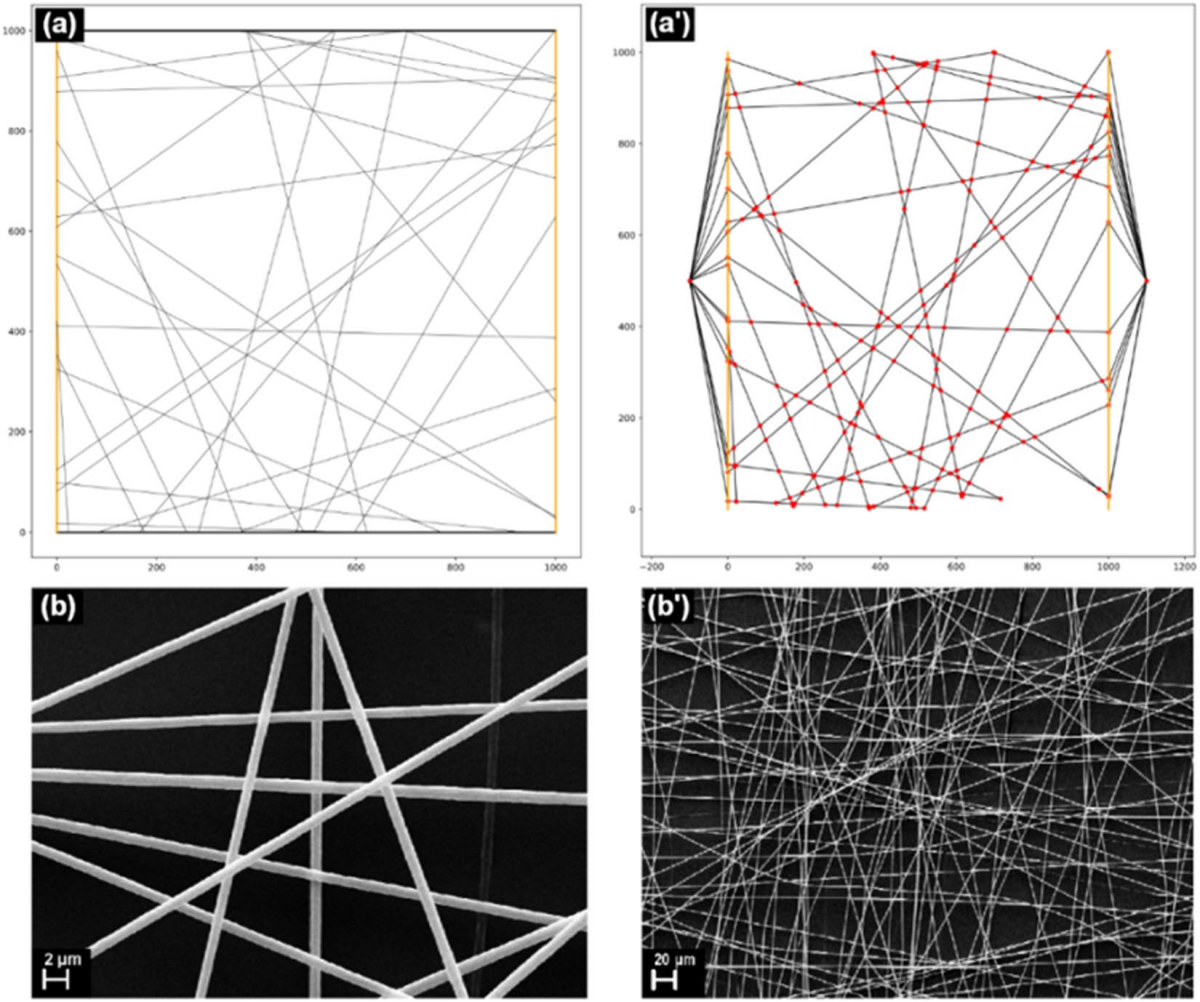
(**a**,a’) The theoretical generated fiber network and the resulting graph; (**b**,b’) SEM images at two magnifications of metalized fiber mat.

A graph is then created from the list of lines and the list of junctions. The latter essentially becomes the list of nodes, while the former is used to create the edges. We assume section area and conductivity to be constant and uniform throughout the web, which leaves length as the only defining variable for an internode segment. As such, each edge is assigned a weight calculated as the geometrical distance between the junctions. To emulate the presence of electrodes, two additional nodes are created and connected via high conductivity edges to all the nodes in two given areas. For a bus bar configuration, the connections are made to all the nodes on the right and left side, respectively. Furthermore, dangling edges are omitted by removing all non-electrode nodes of degree 1. Figure 9(a,a’) shows an example of a generated network and the graph made from it. As well, in Fig. 9(b,b’) is presented the SEM images of the metalized fiber network correlated with the theoretical simulations.

The list of edges, essentially a list of resistors, is extracted from the graph and formatted as a SPICE (Simulation Program with Integrated Circuit Emphasis) netlist, such that it can then be parsed by a circuit simulator. A voltage source node is added and connected to the bus bar nodes and an operating point analysis is run in the simulator. The equivalent resistance of the network can be calculated by using a bus bar configuration and setting the voltage source to 1 V, which is divided by the current passing through the voltage source, yielding the sheet resistance. The circuit simulator software also outputs all the node voltages, which allows the calculation of power dissipation through every segment of the network, by using the equation P = V^2^/R.

The graph data structure was created using the Networkx Python library, while the SciPy Python library package was used throughout the workflow to compute various mathematical functions and perform simple statistical analysis, and visualizations of the simulated webs and graphs were created using Matplotlib^29–31^. Circuit simulations were run in ngspice, an open-source software based on the well-known SPICE^32^.

A 10000 × 10000 simulation area was used to simulate the power distribution in a web with 90% transmission, with applied voltages of 1, 3, 5 and 8 V. To observe the relation between transmission and sheet resistance, 100 different 1000 × 1000 squares were simulated for each transmission value in a given range and their calculated sheet resistances were averaged. Resistivity was fixed at 4·10^−2^ ohm/u.m. in all cases, rounding of values reported in literature for sputtered gold thin films that are over 100 nm thick. Fiber width was fixed at a value of 1 and the thickness at a value of 0.2. Although broad approximations, these values are nevertheless directly comparable to physical samples when the unit for distance is considered to be the micrometer, i.e. the 10000 × 10000 area corresponds to a 1 cm^2^ covered with fibers that have a diameter of 1 µm.

## Electronic supplementary material

Release process

## Data Availability

The datasets generated and analysed during the current study are available from the corresponding author on reasonable request.
